# Lipid vesicle pools studied by passive X-ray microrheology

**DOI:** 10.1140/epje/s10189-023-00375-7

**Published:** 2023-12-07

**Authors:** Titus Czajka, Charlotte Neuhaus, Jette Alfken, Moritz Stammer, Yuriy Chushkin, Diego Pontoni, Christian Hoffmann, Dragomir Milovanovic, Tim Salditt

**Affiliations:** 1 Institut für Röntgenphysik, Georg-August-Universität Göttingen, 37077 Göttingen, Germany; 2 European Synchrotron Radiation Facility, 38043 Grenoble Cedex 9, France; 3 Laboratory of Molecular Neuroscience, German Center for Neurodegenerative Diseases (DZNE), 10117 Berlin, Germany

## Abstract

**Abstract:**

Vesicle pools can form by attractive interaction in a solution, mediated by proteins or divalent ions such as calcium. The pools, which are alternatively also denoted as vesicle clusters, form by liquid-liquid phase separation (LLPS) from an initially homogeneous solution. Due to the short range liquid-like order of vesicles in the pool or cluster, the vesicle-rich phase can also be regarded as a condensate, and one would like to better understand not only the structure of these systems, but also their dynamics. The diffusion of vesicles, in particular, is expected to change when vesicles are arrested in a pool. Here we investigate whether passive microrheology based on X-ray photon correlation spectroscopy (XPCS) is a suitable tool to study model systems of artificial lipid vesicles exhibiting LLPS, and more generally also other heterogeneous biomolecular fluids. We show that by adding highly scattering tracer particles to the solution, valuable information on the single vesicle as well as collective dynamics can be inferred. While the correlation functions reveal freely diffusing tracer particles in solutions at low CaCl$_{2}$ concentrations, the relaxation rate $\Gamma (q)$ shows a nonlinear dependence on $q^2$ at a higher concentration of around 8 mM CaCl$_{2}$, characterised by two linear regimes with a broad cross-over. We explain this finding based on arrested diffusion in percolating vesicle clusters.

**Graphic Abstract:**

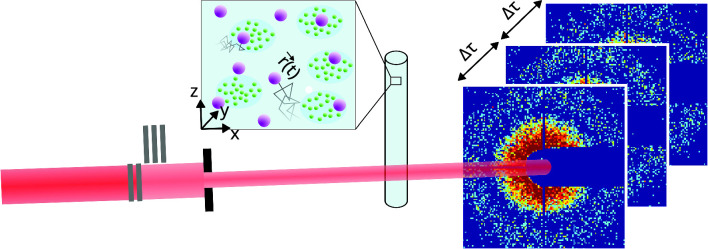

**Supplementary Information:**

The online version contains supplementary material available at 10.1140/epje/s10189-023-00375-7.

## Introduction

Biomolecular fluids exhibit a wide range of still enigmatic properties arising from the complex interplay between structure, dynamics and flow on a multitude of spatiotemporal scales [1]. Viscoelastic properties and molecular mobility are of particular interest, both in equilibrium and in active living systems, for example in protein networks of the cytoskeleton or in membrane assemblies. Apart from theoretical and numerical progress, this field relies on advanced experimental schemes which are compatible with physiologically relevant environments, and which can cover the relevant length and timescales. Further, the available volumes of precious biomolecular samples are typically quite small even for cell-free model systems, making it difficult or impossible to apply classical rheometry. For this reason, passive microscopic observation based on video light microscopy has become a primary research tool for microrheology of biomolecular fluids. By observation of a tracer particle’s trajectory $\textbf{r} (t)$ and the corresponding root mean square (rms) displacement $\sqrt{<{\Delta r}^2 (t)>}$, deviations from free diffusion $\sqrt{6Dt}$ as well as the frequency dependence of the shear modulus $G(\omega )$ can be inferred in light scattering [2], as well as in X-ray scattering experiments [3]. Notwithstanding their importance, dynamic observations based on visible light can also face severe limitations. In dense suspensions, for example, multiple scattering poses particular problems. In other experiments, labelling is difficult or auto-fluorescence compromises signal quality. Finally, at least for samples where fluorescence light microscopy is not possible, such as the analysis of central nerve synapses, the diffraction limit impedes nanoscale resolution [4].

In this work, we explore the suitability of passive microrheology of biomolecular fluids based on X-ray photon correlation spectroscopy (XPCS). In particular, we use colloidal tracer particles to increase the scattering signal while indirectly probing the dynamics of the sample solution or suspension in which the tracer particles diffuse. Diffusion and viscoelastic properties of the biomolecular medium can then be inferred from the analysis of the photon correlation signal as a function of scattering vector *q*. Importantly, boosting the small-angle X-ray scattering (SAXS) signal by the colloidal particles reduces the necessary dose to raise the photon correlation signals above background, which has so far been the central challenge of applying XPCS to biomolecular samples. Previous research has already utilised XPCS experiments with tracer particles [5], although with a main focus on the dynamics in polymers [6–10]. Most recently, the dynamical behaviour of tracer particles was investigated to gain insights into the hardening process of dual-cure (UV/thermal) 3D printing resin [11, 12]. The interaction of the tracer particles can present a concern. Silica nanoparticles, for example, have been shown to adsorb charged lipid vesicles in a buffer solution containing 150 mM NaCl [13]. In practice, one relies on these interactions to be weak enough not to have a strong effect on the diffusion. Only few biological systems have been explored using tracer particles, in particular the interaction between colloids and *E. coli* bacteria [14]. Most notably, however, the interaction between vesicles and $\alpha$-Synuclein has been studied by employing spherical nanoparticle-supported lipid bilayers in place of the vesicles to increase X-ray contrast [15].

XPCS relies on highly coherent synchrotron radiation (SR) or even X-ray free electron lasers (XFEL) as well as advanced detector technology to reach fast timescales necessary for the investigation of biological samples. Various technical progress with respect to detector exposure time [16, 17] and X-ray speckle visibility spectroscopy (XSVS) [18–20] has recently augmented the method to a level where it can be applied to challenging biomolecular samples. Diffusion and transport modes of dense protein solutions, for example, have been studied in seminal XPCS experiments [21–24]. Here, we now want to explore and extend XPCS capabilities in biological complex fluids by adding colloidal tracer particles, which then easily dominate the signal.

As a proof of concept, we study dense suspensions of aggregated lipid vesicle (LV) condensates, which are also denoted as vesicle pools. As a model system, they are considered to recapitulate the reserve pool of synaptic vesicles in the synapse [25]. Clustering of vesicles into aggregates is induced by adding the divalent salt CaCl$_{2}$ which results in strong binding of anionic lipid bilayers, governed by nonlinear electrostatics [26, 27]. Instead of divalent salts, certain proteins such as synapsin can also be used as an “effector protein”, inducing vesicle pool formation by interaction with the vesicular membrane. In fact, synapsin is the most abundant cytosolic protein in the synapses, and known to be associated with pool formation of synaptic vesicles (SV) [28–30]. While the structure of the pools is addressed in an accompanying article [31], this manuscript targets the dynamical properties and in particular the diffusion in suspensions of vesicle pools. However, due to the experimental challenges, this study is exploratory and largely motivated as a technical feasibility study for passive microrheology based on XPCS at low sample concentrations.

The manuscript is organized as follows: After this introduction, experimental methods are presented in Sect. 2, and the main XPCS results in Sect. 3. After the investigation of the dose thresholds for radiation damage, auto-correlation functions are presented for the LV/CaCl$_{2}$ system, with corresponding least-square fits and the *q*-dependence of the model parameters. Two different regimes can be distinguished: At low concentrations of CaCl$_{2}$ we observe short correlation times which we interpret as free diffusion of tracer particles. At higher concentrations and in the sample containing synapsin protein correlation times are three orders of magnitude longer, a sign of arrested motion as tracer particles get trapped in a cluster of vesicles. Two-time correlation functions (TTCF) are then presented for LV/CaCl$_{2}$ and LV/synapsin, exhibiting pronounced non-ergodic behaviour in the case of long correlation times. While the calcium sample shows clear features of growth or arresting motion, the synapsin sample shows a strongly fluctuating TTCF that escapes our current possibilities of detailed interpretation, even at timescales where beam induced damage seems unlikely. A basic numerical toy model is then used in Sect. 4 to study the effects of diffusion with superimposed deterministic movement (e.g. hydrodynamic flow) on the correlation function. This type of motion is likely to occur when tracer particles are trapped in tumbling vesicle clusters but retain some diffusive freedom within the cluster. We find that correlation functions are especially sensitive to flow-induced changes in correlation times at low *q*. Depending on the relative timescales of diffusion and flow, changes with respect to the case of free diffusion are much weaker at high scattering angles, which is in agreement with previous research [32]. The manuscript closes with a discussion section, also summarising the main conclusions.

**Table 1:** Composition of lipid vesicles used for the experiment. Tris buffer contains 25 mM Tris-HCl, 150 mM NaCl and 0.5 mM TCEP and was adjusted to pH 7.4 at 4$^{\circ }$C

Name	Composition				Buffer
	DOPC	DOPS	DOPE	Cholesterol	
LV2	50 %$_{\textrm{mol}}$	50 %$_{\textrm{mol}}$	–	–	H$_{2}$O
LV4	55 %$_{\textrm{mol}}$	20 %$_{\textrm{mol}}$	15 %$_{\textrm{mol}}$	10 %$_{\textrm{mol}}$	Tris buffer

**Fig. 1:**
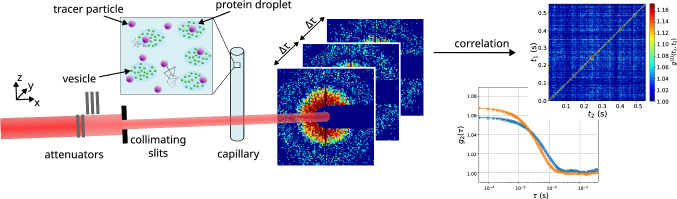
Experimental setup of XPCS experiments. Monochromatic X-rays are attenuated and collimated before encountering the sample. Scattered photons are captured 7 m behind the sample using a 2D-detector. Detector frames are correlated with each other in rings around the beam centre, corresponding to similar magnitudes of the scattering vector. Correlation is performed on GPUs during and after the experiment using the dynamix package. Top left: sketch of the protein sample used for microrheology. Strongly scattering tracer particles are embedded into vesicle-protein clusters

## Methods

### Sample preparation

Two different types of lipid vesicles (LV2, LV4) were prepared at different concentrations. Compositions are shown in Table 1. DOPC (1,2-di-(9Z-octadecenoyl)-sn-glycero-3-phosphocholine), DOPS (1,2-di-(9Z-octadecenoyl)-sn-glycero-3-phospho-L-serine), DOPE (1,2-di-(9Z-octadecenoyl)-sn-glycero-3-phosphoethanolamine) and cholesterol were purchased from Avanti Polar Lipids (Alabaster, AL, USA) in powder form. First, lipid films were produced by mixing stock solutions (10 mg/ml lipids, 2 CHCl$_{3}$: 1 MeOH) and then evaporating the solvent under a stream of N$_{2}$. Residual amounts of liquid were removed by placing the film in a vacuum for at least four hours. Dried lipid films were resuspended with either ultrapure water or a buffer solution (25 mM Tris-HCl, 150 mM NaCl and 0.5 mM TCEP, adjusted to pH 7.4). Vesicles were formed by ten freeze-thaw cycles in liquid nitrogen and a 37$^{\circ }$C water bath. Polydispersity was reduced by passing the vesicle solution through a 100 nm polycarbonate membrane (Avanti Polar Lipids, AL, USA) 21 times. Lipid vesicle concentrations in this paper are given as pre-extrusion concentrations. The expression and purification of synapsin Ia protein is performed as described in [30].

Samples were prepared by mixing vesicle solution with either synapsin Ia protein or calcium chloride to induce cluster formation. For microrheological samples, the sample was dissolved in an equal volume of Duke Standard No. 8050 (Thermo Scientific, Fremont CA, USA) silica colloid solution (diameter $490\pm 20$ nm, 4.1 %$_{\textrm{CV}}$ volume concentration). 30–40 ${\upmu }$l of the mix were filled into a quartz capillary (diameter 1 mm, wall thickness 0.01 mm, Hilgenberg GmbH, Germany) which was sealed with bee wax to limit evaporation of the sample.

### Experimental setup

XPCS experiments were carried out at the EH2 endstation of the ID10 beamline at the ESRF (Grenoble, France). 8.09 keV X-rays were selected from undulator-generated radiation using a Si(111) channel-cut monochromator and adjusted to a beam size of 40x40 ${\upmu \hbox {m}^{2}}$ with high spatial coherence at a flux of $7.32\times 10^{7} \hbox { photons}/\hbox {s}/\upmu \hbox {m}^{2}$. It was measured with a scintillator detector at the nominal uniform-mode beam current of 200 mA. To reduce flux, multiple 80 ${\upmu \hbox {m}}$-thick Si-absorbers could be placed in the beam, resulting in a flux reduction by a factor $T=0.329$ or $T=0.108$ for one or two absorbers, respectively.

Figure 1 illustrates the measurement process. Sample capillaries were placed in a holder mounted on a goniometer head on top of a three-axis diffractometer (Huber Diffraktiontechnik GmbH & Co. KG, Germany). After passing through the sample, the X-rays enter a 7 m-long evacuated flight tube, at the end of which a beamstop blocks the direct beam. Diffraction patterns were recorded on an Eiger500k photon-counting detector (Dectris, Switzerland) at a maximum frame rate of 22 kHz [33]. Key parameters of the setup are summarised in Table 2.

To correlate a sequence of images, every two-dimensional detector frame *I*(*p*, *t*) was separated into 20 rings of width $\Delta q = 0.003\hbox { nm}^{-1}$ starting at $q= 0.0075\hbox { nm}^{-1}$. The intensity autocorrelation function is calculated for each ring according to

1 $$\begin{aligned} g^{(2)}(\tau ,q) = \frac{\langle \langle I(t,p)I(t+\tau ,p)\rangle _p\rangle _t}{\langle \langle I(t,p)\rangle _p\langle I(t+\tau ,p)\rangle _p\rangle _t}~, \end{aligned}$$

where $\langle ...\rangle _p$ denotes the average over all pixels within one ring at radius *q* and $\langle ...\rangle _t$ the temporal average over a sequence of detector images. Additionally, variations over measurement times can be captured with the two-time correlation function (TTCF) $g^{(2)}(t_1,t_2,q)$ that correlates two frames taken at times $t_1$ and $t_2$ with each other according to

2 $$\begin{aligned} g^{(2)}(t_1,t_2,q) = \frac{\langle I(t_1,p)I(t_2,p)\rangle _p}{\langle I(t_1,p)\rangle _p\langle I(t_2,p)\rangle _p}~, \end{aligned}$$

if the measurement was started at time $t=0$.

Processing and correlation of measured data was in large parts performed during the beamtime using a GPU software correlator implemented in the dynamix package (in-house ID10 development together with the ADU of the ESRF).

**Table 2:** Key parameters of the beamline configuration used for the experiment

Parameter	Value
Energy	8.09 keV
Flux @200 mA	$7.32\times 10^{7} \hbox { photons}/\hbox {s}/\upmu \hbox {m}^{2}$
Coherence length (HxV)	$49.6\times {244.1}\,\upmu \hbox {m}^{2}$
Beam size	$40\times {40}\,{\upmu \hbox {m}^{2}}$
Distance sample—detector	7054 mm

## Results

The main challenge of applying XPCS to biomolecular samples is to obtain sufficient signal to noise ratio of the correlation function within the tight limits of dose before radiation damage is observed. This problem is accentuated in biomolecular samples compared to general soft matter, as concentrations of solutions or suspensions are typically low. In order to obtain sufficient signal, the first consideration is partial coherence. Notably, the lateral coherence length $\xi _\perp$ has to be large enough to observe speckle contrast in the far-field diffraction data. This is nowadays fulfilled for beamsizes *d* up to a few tens of microns $\xi _\perp \ge d$ at third and fourth generation synchrotron sources [34]. Secondly, the speckle intensity has to be high enough compared to the background, such that slit scattering, air scattering and sample environment have to receive special consideration. A small difference between sample diffraction and background requires long accumulation times, which then limits the auto-correlation signal to times $\tau \ge \tau _{min}$. In order to reduce $\tau _{min}$, the photon counting error cannot simply be counteracted by increasing the flux or dose rate, for example by tighter focusing, due to the dose constraints. This is even more the case, since damage is a function not only of dose but also of dose rate [35–37]. Hence, a concentration with satisfactory small-angle X-ray scattering (SAXS) signal may not suffice for XPCS.

In the following, we first explore the limits of XPCS for our samples composed of lipid vesicles, buffer and CaCl$_{2}$ or synapsin, in the regime of physiologically relevant concentrations. We show that only by adding signal-enhancing tracer particles, the dynamics of the sample can be captured, much in line with the concept of passive microrheology in optical microscopy. In Sects. 3.2 and 3.3, the analysis of the XPCS-signal is carried out in the fast and slow regimes which can clearly be distinguished, for samples containing lipid vesicles and CaCl$_{2}$ or synapsin Ia protein.

### Signal level, radiation damage, and dose limits

**Fig. 2:**
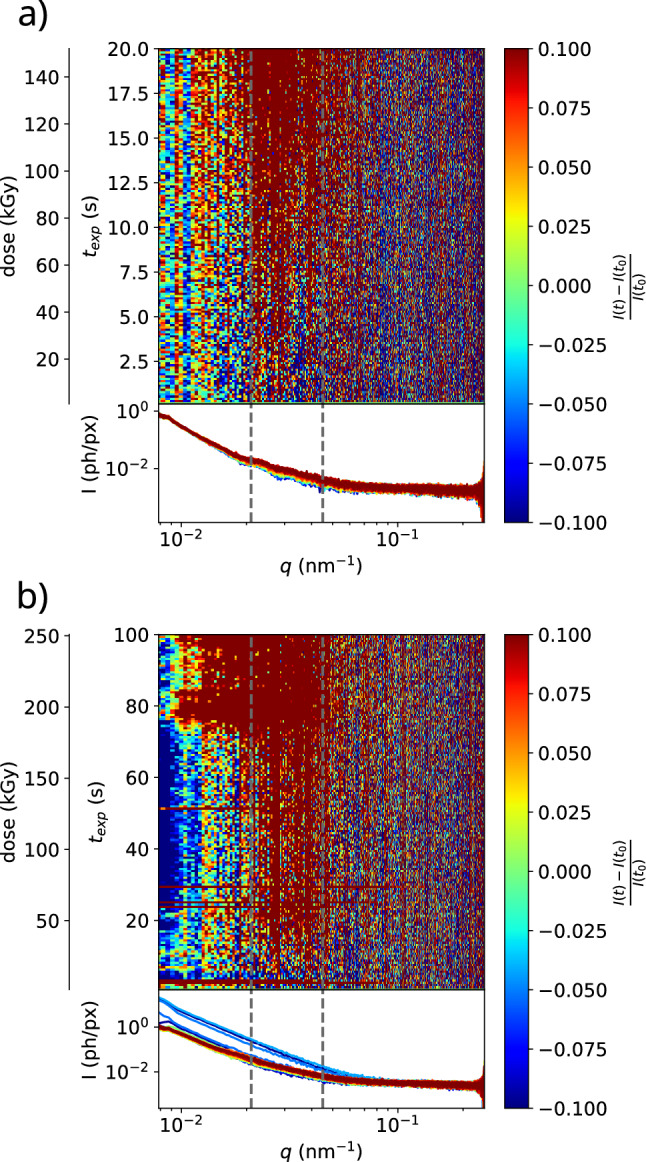
Beam induced effects for samples without tracer particles, exemplified for a sample containing 36 mM LV4 with 3 ${\upmu \hbox {M}}$ Synapsin protein. The diffraction signal is recorded under continuous exposure at different positions along the capillary. a) pristine sample, initial exposure. Beam induced changes (7.5 % relative change in intensity $\Delta I$ in the interval marked by dashed lines) are observed after 17 kGy (at about 2.5 s). The scattering curve is characterised by structure factor effects with small but visible modulations as a function of *q* (no background subtraction). The bottom part shows the radial intensity profiles for consecutive exposure periods. Each trace is averaged over 25 single frames (exposure per frame $t_{exp}={5} \hbox { ms}$). b) After receiving a dose of 152 kGy at 10 different positions, separated by 0.1 mm (beam size: 40 ${\upmu \hbox {m}}$, moving down on the capillary), the signal is not discernible from the background and strong streaks appear already at 6 kGy, indicating that the exposure at previous positions in the capillary may also influence the signal at a new position

Figure 2a exemplifies the challenges of tracer-free XPCS at low concentrations using the example of a mix of 36 mM LV2 and 3 ${\upmu \hbox {M}}$ synapsin in buffer solution. Radiation damage was investigated by successive recording of data at a single spot of the capillary, without moving the sample. Relative changes in the SAXS signal were taken as an indication of radiation damage. Dose calculations were performed as in [36]. The top part in Fig. 2a, shows the relative change of intensity $\Delta I(t) = \left[ I(t=0) - I(t)\right] /I(t=0)$ between the diffraction curves as a function of time *t*, and equivalently the calculated dose $D = t \times \delta$ for given dose rate $\delta = {7.5}\hbox { kGy/s}$. To reduce noise, 25 successive frames were averaged into a single scattering intensity curve. By inspection of $\Delta I (t)$, relative changes in the sample become clearly visible, even when there is little absolute deviation in the intensity. Damage manifests itself predominantly in *q*-intervals, where the absolute scattering intensity displays small deviations from a monotonous decay as these structure-factor related modulations disappear with increasing dose. In the following, an intensity change of 7.5 % with respect to the first intensity profile is used as a threshold criterion for persisting damage to the sample, as such a change implies only minimal damage to the sample but remains clearly discernible from background fluctuations. Accordingly, beam damage is observed after absorbing a dose of 17 kGy, or after exposure periods of about 2.5 s for $\delta = {7.6}\hbox { kGy/s}$. As Figure S1 in the supplementary material indicates, this total exposure time is not sufficient to extract a reasonable decay of the correlation function, i.e. no reliable correlation signal can be deduced. In addition, correlations can be computed at a reasonable noise level only for a few *q*-values in the vicinity of the beam centre because of the low photon count on the detector.

**Fig. 3:**
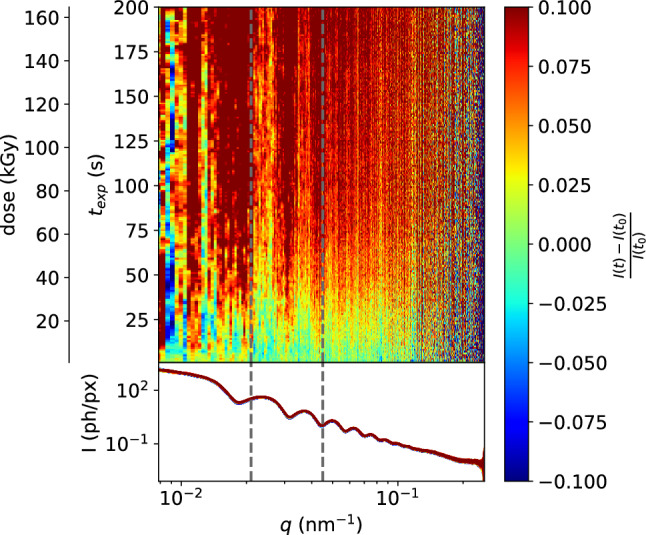
Changes occurring in a sample of 5.4 ${\upmu \hbox {M}}$ Synapsin protein, 3.3 mM LV4 (approx. 1.1 $\%_\textrm{CV}$) and 2.05 ${\%_{\textrm{CV}}}$ tracer particles. Noticeable changes occur at similar doses as in samples without tracer particles, but are limited to narrow *q*-intervals, in contrast to large changes in a broad range before (dose rate 0.82 kGy/s). The 7.5 ${\%_{\textrm{CV}}}$-threshold is reached at 61 kGy in the interval between the two dashed lines. Scattering contrast is much higher now, enabling correlation functions with faster correlation times and for a wider range of *q*-values

To reduce noise, we accumulated signal at different positions along the capillary, and averaged the resulting correlation functions. However, the dose necessary to obtain a strong correlation signal required considerable total accumulation times (including motor movements), over which the beam damage had already spread by diffusion and/or flow in the capillary, and hence a new position could not be assumed to be unaffected, as Fig. 2b illustrates. An analysis of the change of the scattering signal at different positions along the capillary without a change of measurement parameters is provided in Figure S4 in the supplementary material. The damage analysis explained above was carried out again, this time at a lower dose rate $\delta ={2.5} \hbox { kGy/s}$ and after ten consecutive measurements of the type in Fig. 2a, each separated by $\Delta z = {0.1}\hbox { mm}$ along the capillary. As is already visible from the strongly deviating SAXS curves, the sample now undergoes changes right from the beginning of the measurement process. Sudden increases of intensity can tentatively be attributed to bubble formation in the capillary due to previous irradiation at other locations. The first such streak begins at about 1.9 s and many more appear later, whereas in Fig. 2a no such sharp peaks in scattering intensity are visible at all. The initial structure factor variations previously visible at low exposure times were not detected either, despite the increased exposure time of $t_{exp}={25}\hbox { ms}$. Hence, when the signal is sufficient, the dose and dose rate are too high to extract the correlation function $g^{(2)}(\tau )$. If the dose rate is lowered by attenuating the beam intensity, the signal-to-noise is insufficient. Averaging correlation functions measured at different positions along the capillary does not provide a satisfactory solution, due to the spread of beam damage in the capillary, either by diffusion of damaged vesicle assemblies themselves or - more likely - by the damage creating molecular species such as free radicals.

**Fig. 4:**
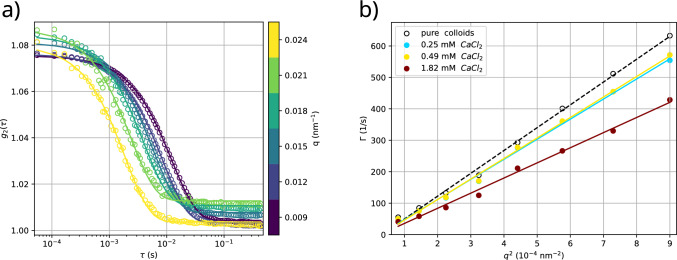
Analysis of calcium-lipsosome clusters with XPCS. a) Example for a sample where the speckles lose correlation after $\tau \approx {\mathcal {O}}(\text {ms})$. Correlation functions for a sample containing 11.9(5) mM LV2 (approx. 3.8 ${\%_{\textrm{CV}}}$), 1.82 mM CaCl$_2$ and 1.94(8) ${\%_{\textrm{CV}}}$ tracer particles. Each set of datapoints corresponds to a specific scattering angle. Correlation functions are averaged over correlation functions from 20 consecutively taken single XPCS image sequences (1.14 kGy at 3.27 kGy/s per sequence). The sample was translated by 0.1 mm along the *z*-direction after each sequence to reduce beam-induced effects on the sample. b) Results of fits for different *q* at different concentrations of CaCl$_{2}$ measured under the same conditions as in a). They indicate a quadratic relationship $\Gamma = D q^2$, an indicator for diffusive behaviour

As a solution to this conundrum, silica colloids were added to the sample to provide a much higher scattering intensity. The dynamic properties of the systems can then be inferred from the movement of the tracer particles, as is commonly done in passive microrheology (e.g. Diffusing-Wave Spectroscopy [38, 39]). Figure 3 shows the beam damage analysis for a sample containing tracer particles. Most importantly, the scattering intensity is now about two orders of magnitude higher than without tracers, making all correlations much more easily accessible even at lower dose. This also provides the possibility of a frame-by-frame correlation to yield a two-time correlation function. In addition, higher scattering intensities provide access to much shorter exposure times, which are now limited only by the maximum detector frame rate of about 22 kHz (cf. next section). Some minor intensity changes are still observed even below 20 kGy at $\delta = {0.82}\hbox { kGy/s}$, but these are now restricted to small intervals, seemingly around the minima of the scattering function. Pronounced changes indicative for untolerable damage appear only much later (7.5 % change in marked range at 61 kGy). Note that we do not expect the tracer particles to change themselves, and as these dominate the scattering signal compared to the vesicle phase, we do not expect large intensity changes, but $\Delta I (t)$ was still found to be a sensitive indicator. Other common criteria such as the positions of maxima or minima [35], or integrated scattering intensities [23], are less well suited when tracer particles are used.

### Fast dynamics

Figure 4a illustrates the dynamics observed at low CaCl$_{2}$ concentrations, where the correlation function decays on the order of milliseconds. This is as expected for freely diffusing tracer particles of the given size in solutions of a viscosity not much different from water. To quantify this, the measured intensity correlation functions were analysed by least-square fits to the Kohlrausch-Williams-Watts (KWW) model function

3 $$\begin{aligned} g^{(2)}(\tau ) = a_0 + a_1 \exp \left( -2(\Gamma \tau )^\beta \right) ~, \end{aligned}$$

where $a_0$ denotes a baseline, $a_1$ contrast, $\Gamma$ relaxation rate and $\beta$ the exponent of decorrelation: $\beta =1$ corresponds to diffusive behaviour, $\beta <1$ yields a stretched exponential, associated with glassy behaviour, and $\beta >1$ implies super-diffusive (e.g. ballistic at $\beta =2$) motion with a compressed exponential [24]. To increase the stability of the fitting process, the KWW exponent was subsequently fixed to $\beta =1.0$, leaving a three-parameter model which assumes free diffusion of the tracer particles. This is justified by the good quality of fit in Fig. 4a. Figure 4b highlights the dependence of $\Gamma$ on the scattering vector *q* for different concentrations of CaCl$_{2}$ up to $c_{{\textrm{CaCl}}_{2}} = {2}\hbox { mM}$.

Assuming normal diffusive behaviour, the relaxation rate $\Gamma$ is related to the diffusion coefficient $D=\Gamma /q^2$. The model can be generalised to account for a collective diffusion coefficient *D*(*q*) that captures the characteristic timescale of movement on different length scales. At low CaCl$_{2}$ concentrations, a constant *D* perfectly accounts for the data, see Fig. 4b. Table 3 presents the corresponding fit values for *D* at different calcium concentrations. The corresponding viscosity calculated from the Stokes-Einstein relation $D = k_B T/6\pi \eta R$ are tabulated as well, calculated for $T={296.15}\hbox { K}$ and $R={250(14)}\hbox { nm}$, which is the radius obtained from a full *q*-range fit to the scattering intensity (see Fig. S2). The resulting viscosity $\eta ={1.22(8)} \hbox { mPas}$ without calcium is larger than for pure water ($\eta = {1.0}\hbox { mPas}$), which can partially be attributed to the high colloid volume fraction in the sample. From pure colloids one would expect an increased viscosity of $\eta _{eff} = \eta (1+2.5\phi ) = {1.1025}\hbox { mPas}$ at a volume fraction of $\phi ={4.1} \,{\%_{\textrm{CV}}}$. The remaining difference can possibly be explained by electrostatic repulsion of the nanoparticles, yielding a larger excluded volume compared to the volume of the scattering particle itself. The addition of vesicles leads to an increase of the viscosity as well, due to an increasing excluded volume. When raising the concentration of CaCl$_{2}$, the viscosity increases further, probably due to the increased volume of vesicle clusters. The rapid decay of $g^{(2)}$ indicates that particles can diffuse freely at low calcium concentration even in the presence of clusters, albeit in a solution of different viscosity. The presence of small clusters does not seem to have a qualitative influence on the diffusive behaviour of tracers.

**Table 3:** Diffusion fits of the relaxation rate $\Gamma$

Sample	*D* ($\upmu \hbox {m}^2$/s)	$\eta$ (mPas)
Pure colloids (no LV)	0.73(2)	1.22(8)
0.25 mM CaCl$_2$	0.64(2)	1.39(9)
0.49 mM CaCl$_2$	0.65(2)	1.36(9)
1.82 mM CaCl$_2$	0.48(2)	1.8(2)

All samples with CaCl$_{2}$ contained 11.9(5) mM LV2 (approx. 3.8 ${\%_{\textrm{CV}}}$) and 1.94(8) ${\%_{\textrm{CV}}}$ tracer particles. Pure tracer particles (Duke particle standard) were measured at 4.1 ${\%_{\textrm{CV}}}$ for comparison. Viscosity $\eta$ was calculated assuming a particle radius $R={250(14)}\hbox { nm}$

### Slow dynamics

**Fig. 5:**
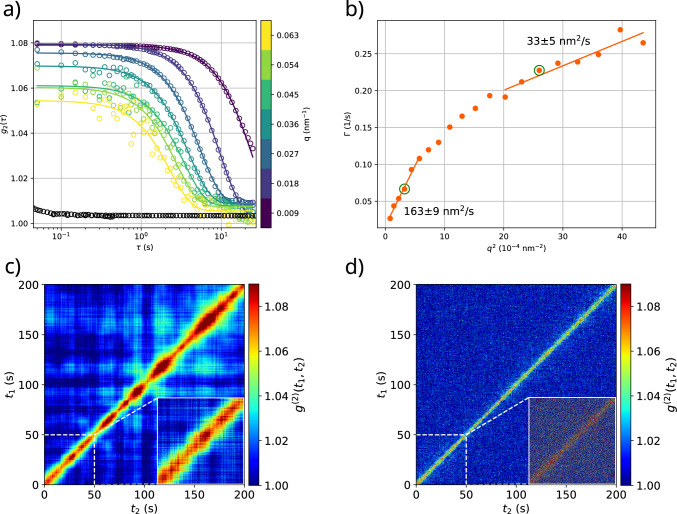
Data and analysis of the sample containing 8 mM CaCl$_{2}$, 10 mM LV2 (approx. 3.2 ${\%_{\textrm{CV}}}$) and 1.64 ${\%_{\textrm{CV}}}$ silica tracer particles (diameter $d={500(28)}\hbox { nm}$ from full-*q* SAXS fit in Fig. S2). a) The correlation decays at times of the order ${\mathcal {O}}(s)$, thus significantly slower than at low calcium concentrations (included as black data for comparison: 1.82 mM CaCl$_{2}$, 11.9(5) mM $\approx$ 3.8 ${\%_{\textrm{CV}}}$ LV2, 1.9 ${\%_{\textrm{CV}}}$ silica particles, $q={0.009} {\hbox { nm}^{-1}}$). b) Fit results showing the *q*-dependence of the inverse correlation time $\Gamma = 1/\tau$ when a KWW function is fitted to the correlation functions (fixed exponent $\beta = 1.6$, details see supplementary material). Two regimes are distinguishable: At low *q*, $\Gamma$ changes at a faster rate than at high *q* ($\Gamma = {0.02}\,{1/\hbox {s}}$ at $q^2=0$). c) and d) Two-time correlation function (TTCF) for the encircled data points in (b), at $q={0.0165}{\hbox { nm}^{-1}}$ and $q={0.0495}{\hbox { nm}^{-1}}$, respectively. A modulation of the width of the diagonal is visible at low scattering angles which seems to disappear at high *q*. White dashed lines indicate the time where significant inhomogeneities start to appear and mark the point up to which the correlation functions in (a) and (b) were calculated

While free diffusion of tracer particles was observed at low calcium concentration, a significant slowing down of the tracer particles was observed at increased calcium concentrations. In a sample containing 8 mM of CaCl$_{2}$, 10 mM LV2 (approx. 3.2 ${\%_{\textrm{CV}}}$) and 1.64 ${\%_{\textrm{CV}}}$ tracer particles, XPCS speckles remain correlated for much longer, with correlation times on the order of ${\mathcal {O}}(\text {s})$. The beam damage appears to be negligible over the period of measurement (200 s at 0.27 kGy/s, see Fig. S6). However, the two-time correlation function (see next paragraph) shows a non-stationary behaviour which sets in after about 50 s, so that the data was only evaluated up to this bound. This reduction of the number of data points which enter in the computation of the correlation function then results in larger errors of the correlation function, in particular at high *q* where the scattering intensity is low. At low *q*, the noise level of $g^{(2)}(\tau )$ is much lower. However, the decay of $g^{(2)}(\tau )$ is not completely captured at low *q*, since $g^{(2)}(\tau )$ does not decay completely over the total evaluated sampling time of 50 s.

As before, a KWW function was fitted to the measured correlation functions for all accessible *q*-values. This time, however, the full model yielded strong fluctuations around $\beta =1.6$, as visualised in Figure S7 in the supplementary material. To avoid overfitting where the correlation function could not be measured completely or where the data are noisy, the KWW-exponent was fixed to $\beta =1.6$ in the fits. Figure 5a presents results of the least-square fits for a selection of *q*-values. For comparison, the correlation curve of the low concentration sample from Fig. 4 is plotted over the same time interval (black circles). The resulting relaxation rate $\Gamma (q)$ are shown in Fig. 5b, exhibiting a nonlinear dependence on $q^2$. In fact, the curve roughly splits into two linear regimes with a broader cross-over. The slope of $\Gamma (q)$ is steep at low *q*-values, corresponding to a large diffusion constant *D*(*q*) at low spatial frequencies, i.e. large length scales. At high *q*, corresponding to high spatial frequencies, *D*(*q*) is smaller. This can be interpreted as slower movement at small length scales and faster, driven motion at large length scales due to trapped tracer particles that follow the tumbling of a vesicle cluster. At the same time, decorrelation at large length scales would not be governed by single-particle diffusion but by collective dynamics due to some kind of hydrodynamic flows. For example, tracer particles attached to a larger cluster can move due to rotation, vibration or tumbling of a cluster (aggregate). As the clusters aggregate, they also start to sediment when getting large enough. At low calcium concentrations, the sedimentation is on the timescale of hours, whereas at high concentrations, such as the 8 mM concentration used here, sedimentation is initially much faster. The process slows down after a few minutes and no changes were observed on the timescale of the measurement times. Note that due to viscoelastic forces, hydrodynamic coupling and boundary conditions, the collective motion can easily become complex, and is likely not in thermodynamic equilibrium. It could, for example, originate from small temperature gradients, floating bubbles or slow sedimentation processes of small clusters.

Importantly, the long correlation time indicates a highly restricted movement of the main scatterers, i.e. the silica tracer particles could be trapped in the vesicle clusters induced by calcium mediated interaction [27].

The two-time correlation function (TTCF) provides further perspective on the microrheological data. Figure 5c displays the development of the correlation between every frame across the total measurement period of 200 s, with each point ($t_1^*$, $t_2^*$) in the chart representing the correlation of the frame captured at time $t_1^*$ with the frame captured at time $t_2^*$. Note that the TTCF is mirrored along the diagonal, as $g^{(2)}(t_1^*, t_2^*) =g^{(2)}(t_2^*, t_1^*)$. The diagonal thus corresponds to the self-correlation, and the (one-time) $g^2(\tau )$ curve is the average along one axis with $\tau$ along the other axis and $\tau =0$ on the diagonal. This representation enables a perspective on the presence and nature of dynamic heterogeneities, ageing or more generally non-stationary and non-ergodic effects. A broadening of the TTCF, as in the case of Fig. 5c indicates the slowing down of the dynamics. Note that the TTCF chart is computed separately for each *q*. The data for $q={0.0165}{\hbox { nm}^{-1}}$, for example, exhibits such a broadening which may be an indication of increased aggregation. In previous work, the partial reappearance of the correlation after some time was attributed to particle growth [40]. For the present case, such an effect could arise when tracer particles which dominate the scattering get trapped in larger vesicle calcium clusters, which might then dissolve again or leave the scattering volume. Note that close to percolation, the cluster or aggregate sizes are not necessarily much smaller than the scattering volume, such that a few entities moving in an out of the scattering volume could easily explain the observed undulatory patterns along the diagonal. The undulations have a characteristic timescale of about 25 s, which increases with time. Such a timescale would correspond to large clusters having a size of about 1.5 mm, a plausible size at the CaCl$_{2}$ concentration of 8 mM. The increase of the characteristic timescale could be explained with the growth of the cluster, as further vesicles are incorporated into the cluster. Interestingly, these manifestations of non-stationary dynamics give way to a more homogeneous behaviour in the TTCFs computed for larger *q*, corresponding to the faster dynamics at small length scales. This can be inferred, for example by comparing the TTCFs for $q={0.0165}{\hbox { nm}^{-1}}$ and $q={0.0495}{\hbox { nm}^{-1}}$, shown in Fig. 5c, d, respectively. The low photon statistics, however, restrict the level of detail visible along the diagonal of the TTCF at higher *q*, see for example the inset in Fig. 5d.

#### Synapsin clusters

**Fig. 6:**
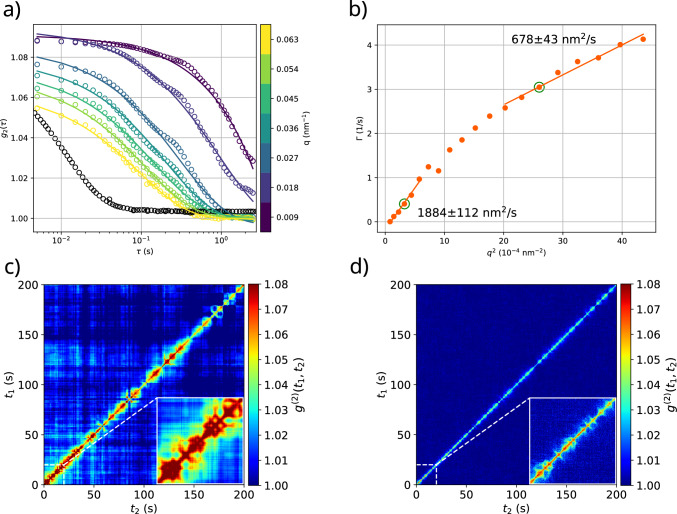
Data and analysis of a synapsin sample containing 5.4 ${\upmu \hbox {M}}$ synapsin Ia, 3.3 mM LV2 (approx. 1.1 ${\%_{\textrm{CV}}}$) and 2.05 ${\%_{\textrm{CV}}}$ tracer particles (protein lipid ratio 1:500). Single KWW fits are shown in a) for different *q*-values and compared against a correlation function of free diffusion (1.82 mM CaCl$_{2}$, 11.9(5) mM $\approx$ 3.8 ${\%_{\textrm{CV}}}$ LV2, 1.9 ${\%_{\textrm{CV}}}$ silica particles, measured at $q={0.009}{\hbox { nm}^{-1}}$ and shown as black circles). Correlation of the detector frames was carried out up to the limit imposed by beamdamage at 25 s. The resulting relaxation rates $\Gamma$ are shown in b) for the case of $\beta =0.7$ fixed. Linear fits assuming the freely diffusive case distinguish two different regimes and yield the effective diffusion constants. c) and d) show the two-time correlations for the encircled fits in b), at $q={0.0165}{\hbox { nm}^{-1}}$ and $q={0.0495}{\hbox { nm}^{-1}}$, respectively. Insets show a magnification of the TTCF at small times, up to the point where beamdamage starts to become visible (cf. supplementary material)

A slowly decaying XPCS correlation was also observed in a microrheological sample containing 5.4 ${\upmu \hbox {M}}$ synapsin Ia protein, 3.3 mM LV4 liposomes (approx. 1.1 ${\%_{\textrm{CV}}}$) and 2.05 ${\%_{\textrm{CV}}}$ silica tracer particles. In contrast to the calcium clusters discussed previously, the dynamics appears to be more complicated. To minimise beam induced effects in the analysis of this presumably radiation sensitive “gel” of lipid vesicles and proteins, correlation functions were calculated only up to a total dose of 20.5 kGy (25 s), in line with the findings of Fig. 3. Figure 6a, b present the corresponding analysis of the one-time correlation function $g^{(2)}(\tau )$, evaluated for the first 25 s. The $g^{(2)}(\tau )$ curves exhibit a two-step decay which becomes gradually less pronounced for increasing *q*. The TTCF, however, indicates that this is not the result of a homogeneous behaviour but rather results from heterogeneous averaging of slow and fast decays, see for example the inset in Fig. 6c. For this reason, we refrained from modelling the $g^2$ decay by a more complicated combination of model functions, but used only a single KWW function to fit $g^{(2)}(\tau )$. In this way we can capture a single characteristic decay time, but avoid over-fitting and opulent model building in a questionable context. For the purpose of a simple and robust fit, which to some extent ignores the two-step decay, the KWW-exponent was fixed to $\beta = 0.7$ (cf. Figure S8). While the correlation decays not as slowly as in the calcium sample of the previous section, the qualitative behaviour (steep at low $q^2$, shallow at high $q^2$) deduced from the KWW fit is quite similar, as Fig. 6b illustrates.

**Fig. 7:**
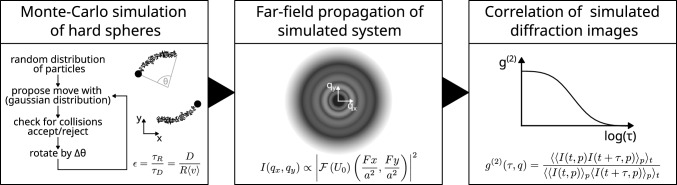
Schematic describing the simulation workflow. First, a Metropolis algorithm generates a sequence of diffusing particles with a superimposed rotational flow. The dimensionless quantity $\epsilon$ provides a relation of the relative strength of the two ways of displacement. The real-space images are then propagated into the far-field by Fourier transformation at a Fresnel number $F=1.48$ and with a pixel size of $a={1}\hbox { px}$. Finally, correlation functions $g^{(2)}(q,\tau )$ and $g^{(2)}(q,t_1,t_2)$ are calculated in the same way, as was done with the experimental data

Stark qualitative differences with the calcium sample arise, however, in the two-time correlations in Fig. 6c, d. Where beam damage is assumed to be negligible (insets), both TTCF display inhomogeneous behaviour, the diagonal widens and narrows with time, indicating complex dynamics of the tracer particles. While in the calcium sample, the TTCF seems to be dominated by a single process, the TTCF for the synapsin sample here is most likely the result of many different dynamical processes. TTCF-based observations and analysis of dynamical heterogeneities have been reported previously [40, 41]. Here, the average correlation time decreases at later times, with bursts of sudden widening and subsequent narrowing of the correlation function along the diagonal. In addition, the bursts become narrower and sparser over time, especially at $q={0.0165}{\hbox { nm}^{-1}}$. Aside from the narrowing of the diagonal, there are also no pronounced qualitative differences between the TTCF at low and high q, as for the Ca-induced clusters. These striking features of the TTCF could possibly be explained by bubble formation or by beam induced dissolution of the synapsin clusters. If a cluster breaks apart, the silica particles could be liberated and would then approach the free diffusive behaviour again. If beamdamage is not a viable explanation (inset), other hydrodynamic influences might be responsible for the observed undulations of the TTCF, as explained in the previous section. They appear on timescales of approximately 5 s in Fig. 6c, which is in agreement with the expected smaller size (300 ${\mu \hbox {m}}$) of large synapsin clusters with respect to calcium-induced clusters. Note, that the synapsin clusters are much smaller and also much more stable than the calcium clusters investigated above. This reduces the influence of size-dependent processes such as sedimentation. A viscoelastic network reacting to hydrodynamic flow could possibly also account for the observations. For this reason, it is instructive to study generic effects of hydrodynamic flow superimposed with diffusion also by simulations, which we address next.

## Simulation

To gain an understanding of how the XPCS data can be interpreted in view of deterministic motion of tracer particles, we perform a simulation conceptually similar to the hydrodynamic flow investigated in [32]. The aim is not to reproduce any particular experimental results but rather to gain more general insights into the interplay between simple deterministic motion and diffusion. As a minimal model, we chose to simulate diffusing spherical particles that rotate around a common centre. The system investigated experimentally in this manuscript could display such a behaviour when tracer particles are constrained in a cluster of vesicles, which itself is subject to hydrodynamic effects, for example advection or sedimentation. These tracer particles are then subjected to the deterministic motion of the cluster, such as tumbling due to sedimentation forces in a crowded environment, in addition to their diffusive motion within the cluster.

To this end, a basic Metropolis Monte-Carlo simulation with superimposed rotational dynamics of the tracer particles was carried out as illustrated in Fig. 7. Fifty particles of diameter $d={40}\hbox { px}$ and uniform density were randomly placed in a thin volume of dimensions 2022 x 2022 x 20 px$^3$ with periodic boundary conditions, approximately resembling the experimental colloid concentration. The simulation is carried out in a pseudo-2D slice, that excludes multiple particles behind each other along the beam direction while at the same time retaining diffusion in three dimensions. Over 200 Metropolis steps (iterations), a shift in the position of each particle is proposed based on a step size drawn from a Gaussian distribution around the current position with standard deviation $\Delta r = {1}\hbox { px}$. The proposition is accepted if the new position does not overlap with another particle. The average step size introduces a timescale based on the law of free diffusion $\langle r^2\rangle \propto D\tau$. Here, we take $\tau _D = {1}\hbox { it}$ and thus $D={1} \hbox { px/it}$. In addition, a rotation by an angle $\Delta \theta = \omega \tau _R$ around the centre of the simulated area is carried out after each step. The rotation was implemented to mimic a non-diffusive flow of tracer particles, for example due to forces imposed by a larger cluster (e.g. tumbling dynamics). At the end of each Metropolis step, the current particle density distribution is projected onto a 2D plane along the short dimension of the simulated volume. The 2D image of the particles is propagated through the sample (complex refractive index $n_{{\textrm{SiO}}_{2}} = 1-7.05\times 10^{-6} + 9.04\times 10^{-8}i$) and further into the (far-field) detector plane (Fresnel number $F = \frac{a^2}{\lambda z} = 1.48$) with a uniform-intensity wave profile.

**Fig. 8:**
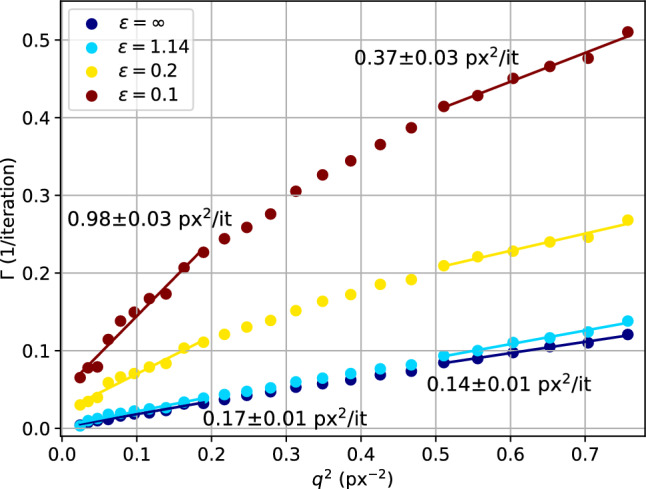
Relaxation rate $\Gamma$ from KWW fits to simulated correlation functions for different $\epsilon = D/R/\langle v \rangle$. Small epsilon denotes faster rotation than diffusion, whereas $\epsilon >1$ represents faster diffusion. $\epsilon =\infty$ corresponds to the case of free diffusion (no rotation). Numbers give the diffusion constants and their errors from linear fits in the regions $q<{0.2}{\hbox { px}^{-2}}$ and $q>{0.5}{\hbox { px}^{-2}}$ for $\epsilon =0.1$ and $\epsilon =\infty$ ($\Gamma _{\epsilon =0.2} = {0.02} \,{1/\hbox {s}}$ and $\Gamma _{\epsilon =0.1} = {0.05}\,{1/\hbox {s}}$ at $q^2=0$)

For the two simulated movements (diffusive and hydrodynamic), the inverse Péclet number $\epsilon = \tau _R/\tau _D = \frac{D}{R\langle v\rangle }$ can be defined for fixed length scales given by the particle radius *R*, relating the timescales over which diffusion and rotation occur. Diffusive and hydrodynamic motion are determined by the diffusion constant *D* and average particle velocity $\langle v\rangle$, respectively. Large $\epsilon$ implies that the movement is dominated by diffusion and small $\epsilon$ indicates dominating rotational (hydrodynamic) effects. Correlation is lost on timescales of the faster process. If $\tau _D$ is smaller than $\tau _R$, diffusion happens faster than rotation, $\epsilon$ is large and the signal decorrelation is governed by a diffusive process and vice versa. At the same time for length scales much larger than *R* or small *q* correspondingly, hydrodynamics should always dominate the decorrelation, even for $\epsilon \ge 1$.

Correlation functions $g^{(2)}(\tau ,q)$ and $g^{(2)}(t_1,t_2,q)$ are then computed using the dynamix package. *q*-dependence is analysed similarly to the experimental data with a bin width $\Delta q = {10}\hbox { px}$ and KWW fit function. Fit results for different values of $\epsilon$ are shown in Fig. 8. When the rotation was switched off completely ($\epsilon =\infty$) or very slow, the random walk of the particles dominates the decorrelation and the gradient $D = \Delta r/6$ corresponds to the mean-squared displacement of a Metropolis step. At lower values of $\epsilon$, the *q*-dependence of the relaxation rate $\Gamma (q)$ exhibits a cross-over from a regime of steeper to flatter slope, similar to the behaviour discussed in Sect. 3.3. At $\epsilon = 0.1$, the average slope for $q<{0.2}{\hbox { px}^{-2}}$ is ${0.98(3)}{\hbox { px}^{2}/\hbox {it}}$ and therefore much higher than the diffusion processes (approx. ${0.17(1)}{\hbox { px}^{2}/\hbox {it}}$). With increasing *q*, however, the gradient seems to converge towards the purely diffusive case, with an average gradient of ${0.37(3)}{\hbox { px}^{2}/\hbox {it}}$.

**Fig. 9:**
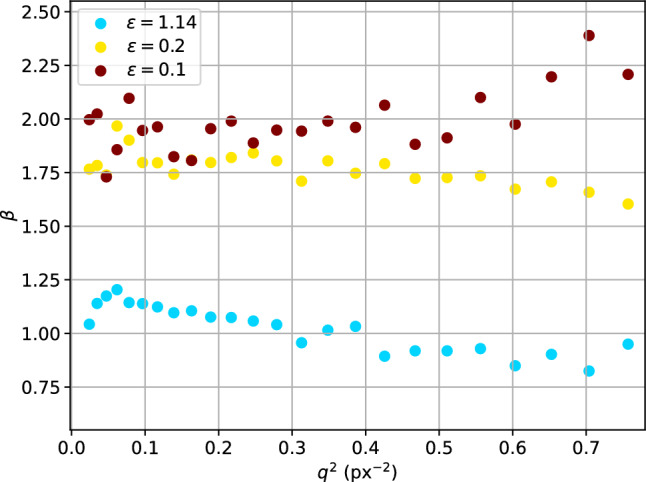
KWW-exponent $\beta$ from fits to simulated correlation functions for different $\epsilon = D/R/\langle v \rangle$. Small epsilon denotes faster rotation than diffusion, whereas $\epsilon >1$ represents faster diffusion. Data for free diffusion ($\epsilon = \infty$) were fixed to $\beta =1.0$ and are omitted here. While dominating diffusion results in exponents close to $\beta =1.0$, fast rotation leads to $\beta =2.0$, indicating ballistic motion

These results can be interpreted from the viewpoint of length scales. Features appearing at low scattering angles correspond to features at large length scales and vice versa. This implies that steep slopes of $\Gamma (q^2)$ indicate fast decorrelation over long distances. Locally (i.e. large *q*), however, the signal remains correlated for longer, implying slow decorrelation. Hence, two points that are separated by a large distance move much faster in relation to each other than two nearby points. As the relative movement of two freely diffusing points should be independent of their separation, the slowest decorrelation of the superpositioned movements should correspond to diffusion in the limiting case. Steeper slopes correspond to the superposition of rotation and diffusion. The smaller $\epsilon$ becomes, the smaller will be the contribution from the diffusing particles.

**Fig. 10:**
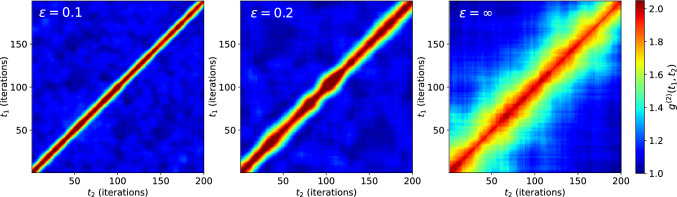
Two-time correlation functions for simulations at three different values of $\epsilon$. TTCFs were calculated at $q={0.2486}{\hbox { px}^{-1}}$, within the region of the low-*q* linear fit of Fig. 8. The TTCFs show increasing width with increasing $\epsilon$ due to longer correlation times as diffusive processes start to dominate the movement. TTCFs appear to fluctuate very little, except when both rotational and diffusive processes contribute to the signal at $\epsilon =0.2$. There, TTCF widens and narrows across the measurement period

Interestingly, the KWW-exponent $\beta$ does not show a similar kink in the *q*-dependence or any indication for two different regimes, see Fig. 9. Aside from residual fluctuations which are likely to be related to the incomplete sampling of the correlation function (cf. supplementary material Fig. S9), the *q*-dependence of $\beta$ is flat. For $\epsilon =1.14$, where diffusion dominates the decorrelation, the KWW-exponent remains close to one, reflecting the diffusive behaviour. At lower $\epsilon$, however, $\beta$ is larger and fluctuates around $\beta =1.75$ and $\beta =2.0$ for $\epsilon =0.2$ and $\epsilon =0.1$, respectively. These values reflect the dominance of the rotation over diffusion in $g^{(2)}(\tau )$. $\beta >1$ is associated with motion which is more directed than diffusion, with $\beta =2.0$ characteristic for ballistic motion, which would be the case for a purely rotational motion of the particles.

Finally, we address the TTCFs, which can also be studied in the simulation framework. The TTCFs exhibit homogeneous behaviour across the measurement period when one of the two processes dominates the correlation function. When rotation is comparatively fast and the motion thus mostly deterministic, the TTCF appears to be almost perfectly straight, in contrast to the diffusive case $\epsilon =\infty$. When both processes contribute to the correlation signal ($\epsilon =0.2$), widening and narrowing of the TTCF is observed, i.e. the hallmark of dynamic heterogeneity.

## Discussion and conclusions

We first address radiation damage and the use of strongly scattering tracer particles to probe dynamics by passive microrheology. In contrast to many other probes, the solution structure of biomolecular assemblies can be readily detected by SAXS. However, while structure analysis may work even for radiation sensitive samples, dynamics by photon correlation is more demanding. For the present case of lipid vesicle condensates, for example, correlation signals required a dose which automatically implied significant damage. To circumvent this problem, we have added passive tracer particles made of silica, which then could either move in solution or associate with a condensate (also denoted as vesicle cluster in this work). Higher scattering intensities now provided access to much shorter exposure times, up to the maximum detector frame rate of about 22 kHz. Data was acquired while translating the capillary, up to a dose of 20 kGy at dose rates of about $\delta = {0.8}\hbox { kGy/s}$. Pronounced changes indicative of intolerable damage appeared only much later (around 60 kGy). These values were calculated without accounting for the movement of the capillary, so that the actual dose was lower since more mass was illuminated. Since free radicals rapidly spread within the capillary, however, a new spot does not imply a pristine sample, and hence the more conservative approach in dose calculation is appropriate. Importantly, within these dose limits, the signal boost of the tracer particles now enabled the assessment of meaningful correlation functions.

At low CaCl$_{2}$ concentrations, the correlation function decayed on the order of milliseconds, as expected for freely diffusing tracer particles of the given size in solutions. A constant *D* perfectly accounted for the data, with moderate decrease of *D* with increasing concentration of CaCl$_{2}$, corresponding to an increase in viscosity due the presence of small (non-percolating) vesicle clusters, which, however, do not change the free diffusion of the tracers. At a concentration of 8 mM CaCl$_{2}$, however, decorrelation of the speckles occurred only on the order of ${\mathcal {O}}(\text {s})$, and the two-time correlation function showed a non-stationary behaviour. The relaxation rate $\Gamma (q)$ showed a nonlinear dependence on $q^2$, characterised by two linear regimes with a broad cross-over, corresponding to a higher effective *D* at low spatial frequencies, and a smaller *D* at high *q*. This can be explained as follows: the tracer particles are now trapped in presumably percolating clusters, moving slowly within the vesicle cluster, accounting for the slow decorrelation on short length scales. At the same time long range structural dynamics, i.e. decorrelation on large length scales happens via hydrodynamic motion of large clusters in solution, which formed by calcium mediated interaction [27]. Similar observations were recently made for percolating networks of protein-RNA condensates [42].

A slowly decaying XPCS signal was also observed in vesicle condensates (clusters) induced by 5.4 ${\upmu \hbox {M}}$ synapsin Ia protein, again characterised by a kink in the $\Gamma (q^2)$ plot, indicative of the two competing processes, first decorrelation by slow diffusion in a cluster, and second by superimposed hydrodynamic flow. In contrast to the calcium induced vesicle aggregates discussed above, the dynamics appeared to be more complicated, with pronounced heterogeneous features in the two-time correlations. Owing to the exploratory nature of the experiments described here, we have not yet carried out a detailed analysis of the complex two-time correlation function of the synapsin sample, regarding for example dynamic heterogeneities as in [43–45]. A viscoelastic network reacting to hydrodynamic flow could possibly account for the observations.

In order to shed more light on how the flow of vesicle clusters might affect the XPCS data, and to confirm the above interpretation, we performed a simple Monte-Carlo simulation of spherical particles with superimposed diffusion and rotation. The respective regimes were classified by the inverse Peclet number $\epsilon = \tau _R/\tau _D = \frac{D}{R\langle v\rangle }$, relating the timescales over which diffusion and rotation occur for fixed length scales given by particle radius *R*. In this basic toy model framework, the experimentally observed kink in $\Gamma (q^2)$ was reproduced. This suggests that hydrodynamic effects on trapped tracer particles play an important part in the explanation of the complex dynamics observed in the experimental XPCS signals. However, further careful experiments and simulations are necessary to pinpoint the exact motion which dominates the observed response. In future, such a combined approach where the generated data is analysed by the same scripts that are used for the experimental data, provides a useful scalable framework to aid the experimental design and interpretation. For example, intended and controlled flow for sample replenishment can be taken into account.

In summary, we have successfully implemented a passive microrheology approach for XPCS studies of biomolecular fluids, in particular vesicle condensates, which are currently under active investigation in the context of liquid-liquid phase separation and as model systems for vesicle pools in the synapse. In this study, we have reached a correlation signal up to a momentum transfer of $q\simeq {0.07}{\hbox { nm}^{-1}}$ corresponding to a (half period) real space distance of about 50 nm, well below the resolution limit of conventional light microscopy. While direct microscopic observation under a light microscope is clearly simpler, tracer-based XPCS of biomolecular assemblies could in particular be used when sample turbidity or thickness is incompatible with optical microscopy. The generalisation to active (for example ATP driven) systems would be particularly interesting.

### Supplementary Information

Below is the link to the electronic supplementary material.

Supplementary file 1 (pdf 2760 KB)

## Data Availability

The datasets generated during and/or analysed during the current study are not publicly available, in part also due to their significant size, but are available from the corresponding author upon reasonable request.
