# Supplementary material for: Lipid vesicle pools studied by passive X-ray microrheology
Source: Eur Phys J E Soft Matter. 2023 Dec 7;46(12):123. doi: 10.1140/epje/s10189-023-00375-7 (PMC10703982; doi:10.1140/epje/s10189-023-00375-7)
Supplement: Supplementary file 1 — (pdf 2760 KB) [file 10189_2023_375_MOESM1_ESM.pdf]

Supplementary Material to "Lipid vesicle pools studied by passive x-ray microrheology"

Titus Czajka[1], Charlotte Neuhaus[1], Jette Alfken[1], Moritz Stammer[1], Yuriy Chushkin[2], Diego Pontoni[2], Christian Hoffmann[3], Dragomir Milovanovic[3], Tim Salditt[1*]

[1*]Institut für Röntgenphysik, Georg-August-Universität Göttingen, 37077 Göttingen, Germany.
[2]ESRF, The European Synchrotron, 38043 Grenoble Cedex 9, France.
[3]Laboratory of Molecular Neuroscience, German Center for Neurodegenerative Diseases (DZNE), 10117 Berlin, Germany.

*Corresponding author(s). E-mail(s): tsaldit@gwdg.de;

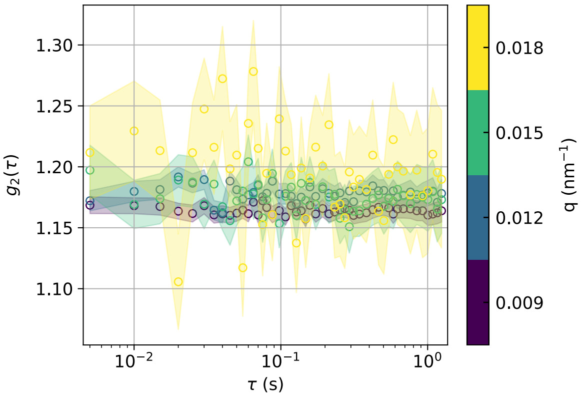

Supplementary Figure S1 The correlation function remains flat for the data of Figure 2a, analysed for a time (dose) range where the scattering intensity remains unchanged (up to 2.5 s total measurement time). Only few q-values are accessible for correlation at a reasonable noise level due to the low scattering signal of the sample. The speckle contrast is at about 1.17 and above the observed speckle contrast, thus indicating a correlation of residual scatterers due to low signal to noise ratio.

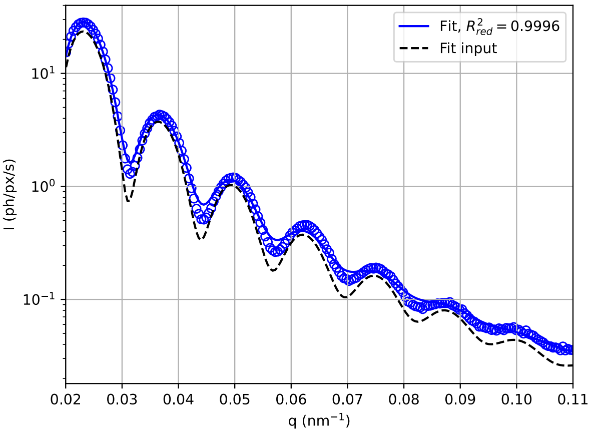

Supplementary Figure S2 Fit of the SAXS intensity distribution for a sample of silica tracer particles at 4.1 $\%_{\mathrm{CV}}$ with $r = 245\,\mathrm{nm}$. The fit model consists of a Gaussian distribution of spheres with different radii. Results: $r = 249.96\,\mathrm{nm}$, $dr = 13.7\,\mathrm{nm}$

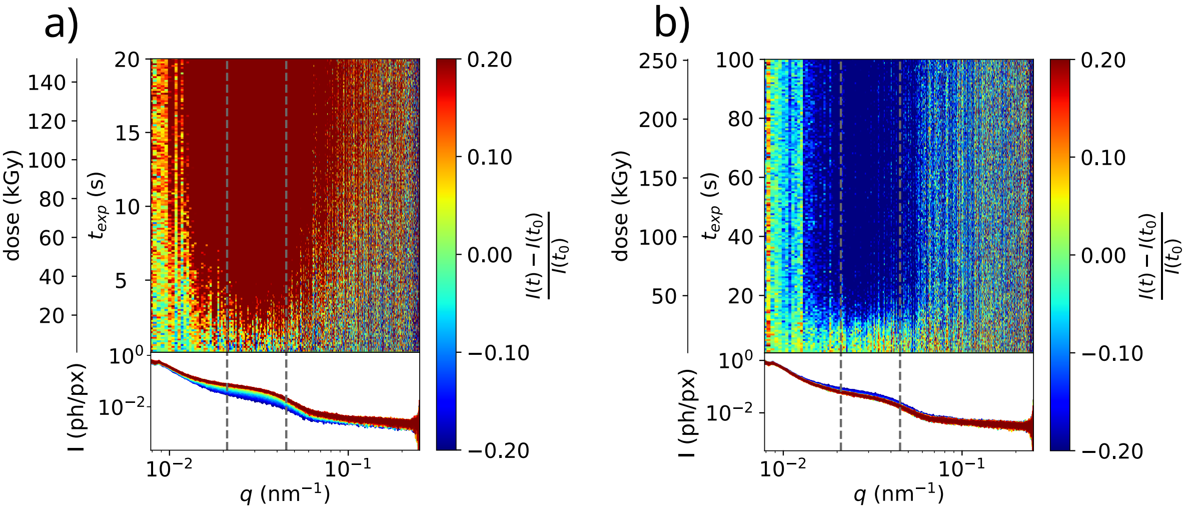

Supplementary Figure S3 Changes in a 50 mM LV2, 4 mM $CaCl_2$ sample under continuous exposure at different positions along the capillary. a) Unmeasured sample, initial exposure. Beamdamage appears after 9 kGy (about 1.5 s, the SAXS signal has a structure factor that increases with increasing exposure (no background subtraction). b) After having been exposed to 152 kGy at 5 different positions, separated by 0.1 mm (beam size: 40 µm, moving down on the capillary), the signal strength is now decreasing with increasing exposure and the 7.5 % beam damage threshold is crossed after 25 kGy (ca. 10 s).

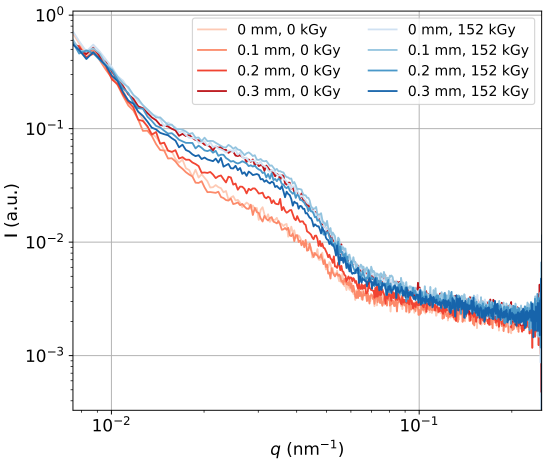

Supplementary Figure S4 Illustration of the spread of beamdamage along a capillary using the same sample as in Fig. S3. Initially, during the first measurement of the capillary, heavy beamdamage occurs with an increase of scattering signal at intermediary q-values (cf. also Fig. S3a) as the sample absorbs a radiation dose of 152 kGy. A second measurement, 0.1 mm below the initial measurement position, displays a very similar behaviour. This supports the hypothesis that the sample is homogenous. During the third measurement, 0.2 mm below the initial measurement position, the scattering intensity converges with increasing dose towards the same intensity distribution as before. The qualitative and limiting behaviour indicates that the sample composition is similar to the one observed in the first measurement and that the initial difference at 0 kGy is a result of a spread of the damage along the capillary. In a fourth measurement, 0.3 mm below the first measurement position, the initial damage to the sample at that position is already so large, that it appears as damaged as sample during the final measurements on the previous spots. The qualitative change during further exposure is then different, as can also be seen in Fig. S3b.

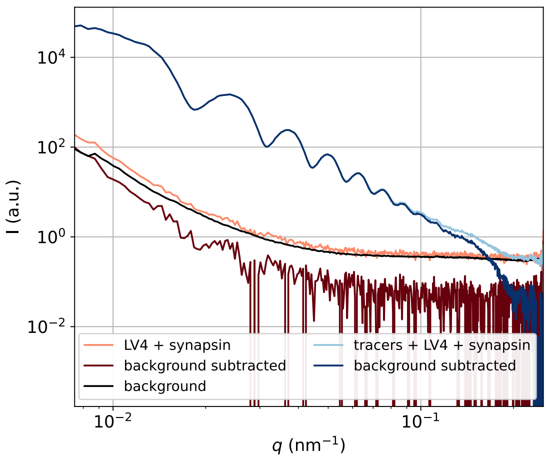

Supplementary Figure S5 Comparison of SAXS measurements with and without background subtraction of the samples discussed in Section 3.1. In samples without tracer particles, the signal is only barely distinguishable from the background and would require tuning of the background level, according to the $CaCl_2$ concentration and the presence of buffer. When tracer particles are visible, however, the signal is much stronger and only (visibly) influenced at very high q, where the signal is not used due to high noise levels at very fast exposure times. As the main interest of the manuscript is on the dynamic signals, we chose to work without background correction. Additionally, background subtraction might not be justified in the presence of correlations.

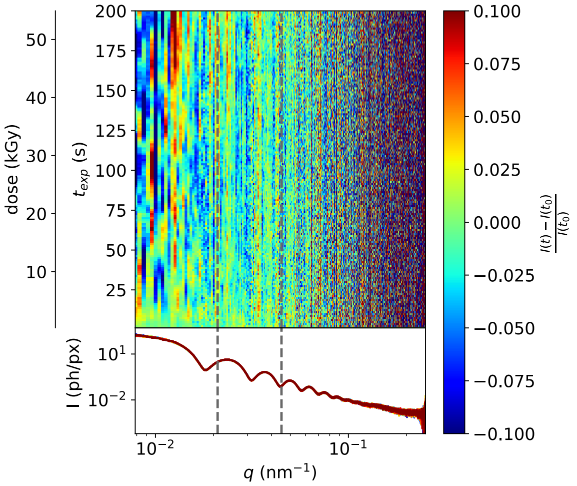

Supplementary Figure S6 Beam damage analysis of the sample discussed in Section 3.3. Sample contains 4 mM $CaCl_2$, 12 mM LV2 and 2.1 $\%_{CV}$ tracer particles. At a dose rate of 0.27 kGy/s, no significant changes of relative scattering intensity ΔI can be seen. Thus, the sample is assumed to be free of beam induced damage.

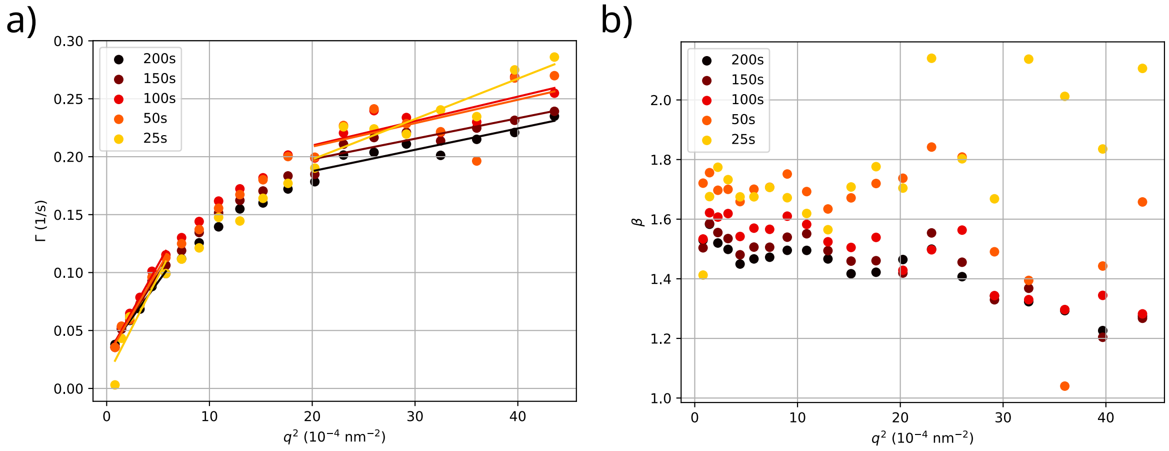

Supplementary Figure S7 Free KWW fit for sample in Figure 5 of the article.

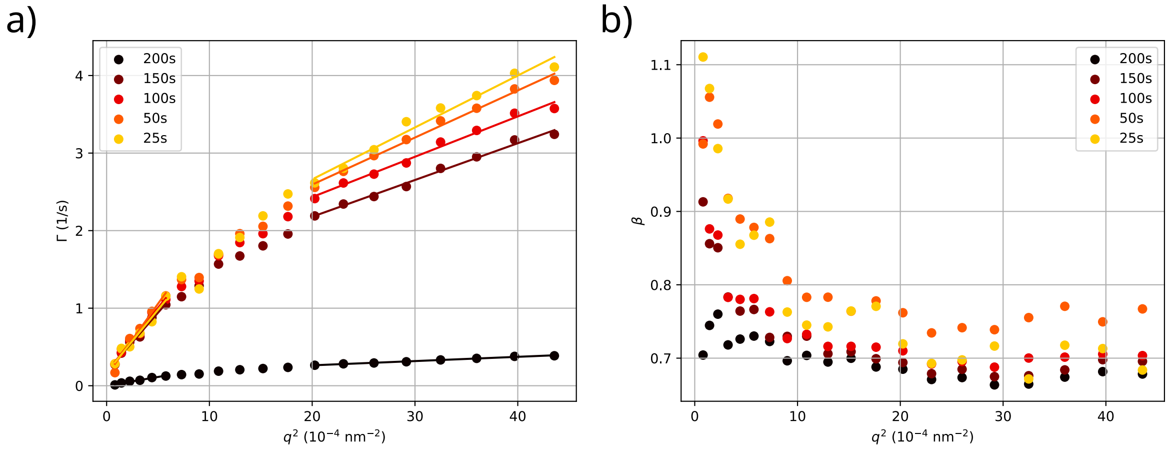

Supplementary Figure S8 Free KWW fit for synapsin sample in Figure 6 of the article.

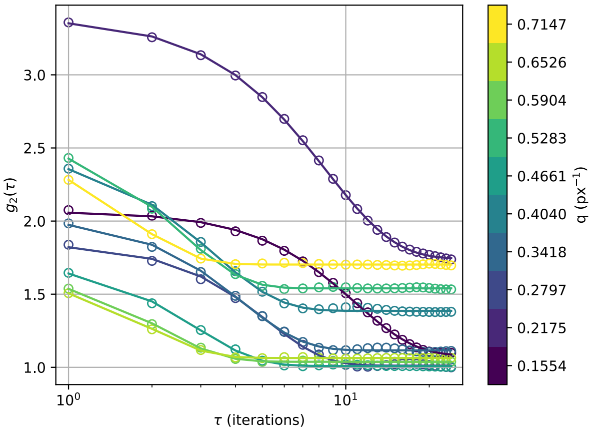

Supplementary Figure S9 Correlation function $g^{(2)}(\tau)$ of a simulated colloid system with $\epsilon = 0.1$.
